# Bioremediation efficacy—comparison of nutrient removal from an anaerobic digest waste-based medium by an algal consortium before and after cryopreservation

**DOI:** 10.1007/s10811-017-1066-x

**Published:** 2017-02-05

**Authors:** Alla Silkina, Graham D. Nelson, Catherine E. Bayliss, Craig L. Pooley, John G. Day

**Affiliations:** 1 Centre for Sustainable Aquatic Research (CSAR), Swansea University, Swansea, SA2 8PP UK; 2 The Culture Collection for Algae and Protozoa, Scottish Association for Marine Science, Scottish Marine Institute, Oban, PA37 1QA UK

**Keywords:** Alga, Algal consortium, Bioremediation, Cryopreservation

## Abstract

An algal consortium was isolated from an integrated steelmaking site at TATA Steel Strip Products Ltd. in Port Talbot, UK, and its bioremediation capacity tested. Excellent “bioremediation” was observed when the mixed culture was “applied” to diluted effluent from an enhanced anaerobic digestion plant at Dŵr Cymru Welsh Water at Port Talbot, UK. After 5 days of cultivation in a 600-L photobioreactor, 99% of the total nitrogen (initial level, 4500 μmol L^−1^) and total phosphorus (initial level, 690.4 μmol L^−1^) were removed from the waste stream. The consortium was deposited in the Culture Collection of Algae and Protozoa (CCAP), an international depository authority for microalgal patents, as CCAP 293/1. This material has been successfully cryopreserved using a two-step cryopreservation protocol with dimethyl sulphoxide (5% *v*/*v*) used as a cryoprotectant. On recovery of samples after 3 months storage at −196 °C, the specific bioremediation activity of the revived consortium was tested. The capacity of the revived culture to bioremediate effluent was not significantly different (*p* < 0.05) from a non-cryopreserved control, with 99% of total nitrogen and phosphorus remediated by day 4. Although non-axenic algal cultures have previously been cryopreserved, this is the first report of the successful cryopreservation of mixed algal consortium, with validation of its ability to bioremediate after thawing comparing non-cryopreserved cultures with a revived post-thaw algal consortium. The study also highlights the need to ensure the long-term security and the requirement to validate the functionality of conserved inocula with biotechnological/bioremediation potential.

## Introduction

There is a long tradition of bioremediation of waste streams by microalgae with early work undertaken by Oswald and colleagues in the 1950s (Oswald et al. 1953a, b, 1957b) leading to the subsequent widespread use of high rate algal ponds (HRAPs) for effluent treatment in many countries. In addition to the capacity of algae in HRAPs to utilise nutrients, including dissolved nitrates and phosphates, some taxa can degrade toxicants such as high molecular weight polycyclic aromatic hydrocarbons (Juhasz and Naidu 2000). Furthermore, algae can adsorb heavy metals and other toxicants in aquatic environments (Gadd 2009). Current technology for algal wastewater treatment uses HRAPs; however, low algal productivity (generally ∼10 t ha^−1^ year^−1^) and requirement for expensive processes for algal harvest limit the commercial exploitation of algal biomass (Craggs et al. 2011). With the development of novel technologies, this process has become more efficient and cost effective, for example the use of shallow, paddlewheel-mixed, HRAPs can result in productivities >30 t ha^−1^year^−1^ and better consistency of nutrient removal (Craggs et al. 2011). Furthermore, considerable amounts of research have been focused on the development of photobioreactors (PBRs) over the past two decades and a wide range of different designs and configurations have been built and tested around the world (Pulz 2001; Singh and Sharma 2012). Successful PBR cultivation systems, with high biomass productivity, have been demonstrated in high latitudes (Fuentes-Gruenewald et al. 2015). These have used semi-continuous cultivation approaches with resultant high nutrient removal rates employing *Porphyridium purpureum* and *Scenedsmus* sp. (Ruiz-Marin et al. 2010).

The waste material from an anaerobic digestion (AD) plant was used in this study. This effluent contains high concentrations of nitrogen (in the form of ammonia) and phosphorus and may cause environmental problems if discharged without treatment (Li et al. 2011). The reduction of nutrient levels in effluent is required both to avoid environmental damage on discharge and to conform to the local and domestic legal requirements (Welsh Government DfEFaRA 2014). These requirements can be performed by microalgae because of their capacity to use nitrogen and phosphorus in their metabolism (Pittman et al. 2011). However, the efficiency of the algal bioremediation process primarily depends on the algal strain selected or the constituents of a microbial consortium involved. To date, the majority of studies that have focused on remediation have utilised uni-algal cultures (Wang et al. 2010; Lizzul et al. 2014; Praveenkumar et al. 2014; Whitton et al. 2016; Schulze et al. 2017). The use of algal consortia has been less extensively studied, although a number of recent reports have indicated that this strategy can outperform pure culture applications (Dalrymple et al. 2013; Samorì et al. 2013).

A key aspect of this study has been the use of a mixed algal bacterial consortium from a polluted ecosystem, which was the basis of a patent submission (Silkina and Nelson 2014). The stability and sustainability of mixed cultures has previously been reported (Kumar and Goyal 2009; Van Den Hende 2014; Van Den Hende et al. 2014a, 2014b, 2015, 2016). Furthermore, isolated mixed consortia from polluted ecosystems have already adapted to survive on the waste stream as the natural selection process has already been undertaken. Thus, there is a high probability that the consortium will be more stable than an artificially formulated algal/bacterial mixture. Additionally, due to the high level of tolerance of pollutants, it is probable that mixed consortia should acclimate more quickly than a monoculture culture. Studied mixed consortia composed of tolerant species, most commonly chlorophytes, have previously been reported for their capacity to remediate waste water (Khan et al. 2008; Ruiz-Marin et al. 2010; Wang et al.^2010^). In these studies, the mixed consortia had higher rates of uptake of nitrogen and phosphorous, as the different species could utilise different uptake mechanisms. Algae are known to have mechanisms for the uptake of various forms of nitrogen, namely, ammonia, nitrate and amino acids; these are highly variable within the algal community and individual algal strains may have differing nutrient uptake capacities (Olguın 2003; Barsanti and Gualtieri 2006; Cai et al. 2013). The growth of the mixed consortia results in a rapid increase in pH of the growing culture. Under this condition, in addition to the algal uptake, phosphorus precipitation enhances the remediation process (Laliberte et al. 1997). A further advantage is that a mixed consortium has better ability to uptake carbon, as oxygen evolution by the algae facilitates aerobic bacterial growth and in addition many of the algae involved can directly sequester carbon via mixotrophic, or heterotrophic, growth (Oswald et al. 1957a; Day and Turner 1992).

The maintenance of a functionally stable, reproducible master stock culture, in this case, an algal consortium, is a pre-requisite for sustainable remediation, as it is in any other biotechnological process employing microorganisms. Furthermore, the *ex situ* maintenance and deposition of the microorganism(s) in an international depository authority (IDA) under the terms of the Budapest Treaty (Anonymous 1977) are absolute requirements on submission of a patent. This raises specific challenges as IDAs are contracted to maintain the organisms, without loss of their desired patent-related characteristics for in excess of 15 years. Whilst for some algae, serial transfer may suffice and stability of metabolite production may be maintained, as in the case of pigment mutants of *Parachlorella kesslerii* (Müller et al. 2007), for other algal taxa, it may result in loss of key characters/capabilities (Day and Fleck 2015). Serial transfer cannot absolutely guarantee retention of key characteristics, and although there are no published data on the retention or loss of bioremediation capability of algal consortia that have been held under laboratory conditions for extensive periods, alternative conservation strategies that do not require maintaining growing cultures may be optimal. In addition to the possibility of loss or reduction in efficacy of the characteristics relevant to the patent application, serial transfer by its nature has the potential to result in human-error-induced mistakes such as mislabelling or contamination on transfer. Therefore, in addition to issues associated with functional stability, alternative long-term preservation methods that minimise handling/manipulation are needed to guarantee that materials remain “fit for purpose”. Cryopreservation, storage at ultra-low temperatures (normally −80 °C for prokaryotes and <−120 °C for eukaryotes), is widely accepted as the optimal method for the conservation of patented strains, although for many bacterial taxa, freeze-drying (lyophilisation) may also be applicable (Day and Stacey 2008).

Generally, algal cultures held in research laboratories, culture collections or by commercial organisations for biotechnological applications are maintained as uni-algal (monocultures) but not necessarily axenic cultures (Lorenz et al. 2005). Furthermore, the commensal bacteria may be vital to maintaining healthy functional cultures (Prakash et al. 2011; Amaral et al. 2013). Cryopreservation has been widely employed to maintain algal cultures; however, for the majority of the protocols employed, axenicity has been the key to successful recovery of a healthy algal culture (Taylor and Fletcher 1998; Day and Fleck 2015). Where cryopreservation has been applied to non-axenic algal cultures, in some cases, additional procedural steps may be required to reduce available carbon released as a result of cell lysis that may result in a bacterial “bloom” and subsequent algal death (Heesch et al. 2012; Amaral et al. 2013). This study explored a scenario where there was a functionally stable, mixed, algal-bacterial flora. The objective was to demonstrate that cryopreservation could be used to conserve this undefined algal consortium and to validate that the application of a standard cryopreservation approach could be employed to ensure functional stability of the consortium.

## Material and methods

### Algal consortium and cultivation

The ACCOMPLISH algal consortium was isolated by taking water samples from an integrated steelmaking site in spring 2012 (Port Talbot, UK) and cultivating these on a defined freshwater algal medium based on the nutrient levels employed in f/2 medium (Guillard 1975). The most robust consortium, i.e. where no obvious change in the algal mix was observed on successive transfers, has been used in a bioremediation study using nutrient media based on the waste stream from an enhanced anaerobic digestion plant (Port Talbot, UK) (Silkina et al. 2015). Since February 2012 this consortium has been maintained in the Bioscience Department microalgal culture collection (Swansea) in a freshwater algal medium based on the f/2 nutrient profile (Guillard 1975) under 18 °C, 100 μmol photons m^−2^ s^−1^ with 16:8 light/dark cycle. The material was deposited in the Culture Collection of Algae and Protozoa as a patent deposit and allocated the unique identifier CCAP 293/1 (note: patent strains are not in the public domain and cannot be directly obtained from the CCAP).

The predominant algal species in the consortium was identified by Banco Espanol de Algaes (BEA), Grand Canarias, Spain, as *Franceia amphitricha.* Other algal taxa present in the consortium included *Scenedesmus* sp., *Chlorella* sp., *Chlamydomonas* sp. and *Desmodesmus* sp. (Table 1).

**Table 1:** Taxonomic designation of algal taxa in CCAP 293/1 consortium

Species	Family	Class
*Franceia amphitricha*	Oocystaceae	Trebouxiophyceae
*Scenedesmus* sp.	Scenedesmaceae	Chlorophyceae
*Chlorella* sp.	Chlorellaceae	Trebouxiophyceae
*Chlamydomonas* sp.	Chlamydomonadaceae	Chlorophyceae
*Desmodesmus* sp.	Scenedesmaceae	Chlorophyceae

### Bioremediation assessment

The control medium employed was a freshwater algal medium based on the nutrient levels employed in f/2 medium (Guillard 1975). The waste-base nutrient medium was prepared using Dwr Cymru Welsh Water’s (DCWW) anaerobic digested (AD) effluent sampled directly after the digester gravity belt filters.

Fresh samples of DCWW’s AD waste effluent were stored in a fridge at 4 °C before being treated. The samples had high levels of turbidity and were passed through a 100-μm bag filter and then filtered through a 0.2-μm hollow fibre cartridge (GE Healthcare, USA) to improve the effluent’s clarity. To assess optical density, a direct light measurement was selected in the NIR (near-infrared) spectrum, at a wavelength of 750 nm, so that colour influences would not affect the optical density values. The supernatant was then autoclaved at 121 °C for 20 min and, after cooling, stored in hermetically sealed containers at 4 °C for no longer than 1 month. Dilutions of this material to final concentrations of 1, 5 and 10% (*v*/*v*) were made to test their suitability in media formulations.

A bench scale trial was conducted using 2-L flasks externally illuminated on one side by a twin florescent tube (Natural daylight Osram tube). Each flask was sealed using a nitrile rubber bung with two separate holes drilled for glass tubes to be inserted, one for aeration and sampling and the other for venting the exhaust gas. The room temperature was maintained at 18.0 ± 3 °C. Lighting from the florescent tubes was provided by an 18:6 light cycle at 200 μmol photons m^−2^ s^−1^ with samples taken at the end of the light cycle. The flask was continuously sparged with ambient air at 0.1 vvm, with the addition of 0.03% (*v*/*v*) CO_2_ during the light cycle. The pH was maintained by the addition of 10 mg L^−1^ sodium bicarbonate.

Daily samples (15 mL) were aseptically taken from the cultures, their pH immediately measured, then cell concentration and biovolume were assessed by Coulter Counter (Multisizer 4). Samples for water chemistry analysis were taken every 24 h and centrifuged for 15 min at 3000*×g*. Supernatant samples were then passed through GF/F Whatman filters and stored frozen at −20 °C for 1 week prior to analysis.

Scale-up cultivation experiments were performed using two 600-L capacity horizontal tubular photobioreactors (BioFence, from Varicon Aqua Solution manufacturing) located in a heated greenhouse, Swansea, UK. The duration of experiment was a 12-day consecutive period. The abiotic conditions were temperature 18–25 °C and 2% CO_2_ injection, which was regulated by pH measurements (i.e. CO_2_ addition when the medium was above pH 7.5). The natural (greenhouse) light conditions were variable over the experimental period ranging from 300 to 1000 μmol photons m^−2^ s^−1^, with a light/dark cycle of 16:8 h. During the cultivation period, waste-based medium [10% (*v*/*v*), 100 mL of prepared waste solution for 1 L of culture] was tested against a control f/2 medium.

## Sample analyses

### Cell growth

Every 24 h, cell concentration, cell size and biovolume measurements were performed by Coulter Counter Multisizer 4, Beckman, USA, to quantify culture growth as described by (Mayers et al. 2013).

The growth rate was calculated based on biovolume using the formula provided by Levasseur et al. (1993):

$$ \mathrm{Growth}\ \mathrm{rate}: K\hbox{'}=\mathrm{Ln}\left({N}_2/{N}_1\right)/\left({t}_2-{t}_1\right) $$

where *N*_*1*_ and *N*_*2*_ = biovolume (measured by Coulter counter) at time 1 (*t*_1_) and time 2 (*t*_2_), respectively.

### Remediation N and P uptake

The nutrient levels in the algal media were analysed every other day using an automated segmented flow analyser (AA3, Bran Luebbe, Germany). The automated procedure for the determination of nitrate (NO_3_) and nitrite (NO_2_) uses the process whereby nitrate is reduced to nitrite by a copper-cadmium reductor column (Armstrong et al. 1967). The nitrite then reacts with sulfanilamide under acidic conditions to form a diazo compound. This compound then couples with *N*-1-naphthylethylene diamine dihydrochloride to form a purple azo dye. Nitrate/nitrite analysis was performed, which along with ammonia, measured by the Berthelot reaction, in which a blue-green coloured complex is formed that is measured at 660 nm, gave total nitrogen (TN). The automated procedure for the determination of ortho-phosphate is based on the colorimetric method in which a blue colour is formed by the reaction of ortho-phosphate, molybdate ion and antimony ion followed by reduction with ascorbic acid at a pH < 1. The reduced blue phospho-molybdenum complex is read at 880 nm.

### Cryopreservation of the algal consortium

Cryopreservation was performed according to Day and DeVille (1995). Dimethyl sulphoxide (DMSO) (Sigma-Aldrich Ltd., UK) was filter sterilised in a sterile f/2 freshwater algal medium to a final concentration of 10% (*v*/*v*) using a 0.20-μm sterile syringe filter (Iwaki, Japan). An aliquot (10 mL) of the 10% (*v*/*v*) DMSO solution was aseptically added to 10 mL of an early stationary phase culture of the algal consortium CCAP 293/1 in a sterilised Universal bottle (25 mL). The Universal was inverted several times to ensure complete mixing and the algal culture in 5% (*v*/*v*). DMSO was dispensed in 1.0 mL aliquots into cryovials (Greiner Bio-One GmbH, Germany). These were then incubated at room temperature (∼20 °C) for 20 min prior to cryopreservation to enable the cryoprotectant to enter the cells. The cryovials were then transferred to a controlled rate cooler (Kryo 360 3.3, Planer plc, UK). The samples were cooled at −1 °C min^−1^ between 20 and −40 °C, with auto-ice nucleation at −5 °C, and then held for a further 15 min at −40 °C. The cryovials were then rapidly removed from the cooler unit, plunged into liquid nitrogen (LN_2_) and transferred to the CCAP cryobank for storage in liquid phase liquid nitrogen (−196 °C).

After 24 h, and again after 3 months, storage triplicate samples were transferred in LN_2_ from the cryostorage facility to the lab. They were then thawed by direct immersion in a preheated water bath at 40 °C and were removed as soon as all visible ice had melted. Immediately after thawing, the samples were aseptically inoculated into tissue culture flasks containing 20 mL of the sterile f/2 freshwater medium. The flasks were wrapped in aluminium foil to prevent the possibility of light-induced stresses/metabolic uncoupling and incubated at 20 °C in the dark for 36 h before removal of the aluminium. For culture regrowth, samples were incubated under a 12:12 h light/dark regime, irradiance ∼30 μmol photons m^−2^ s^−1^ PAR for 3 weeks and periodically visually assessed and examined by phase contrast microscopy to confirm regrowth. The cultures (after cryopreservation) derived from samples which had been stored for 3 months were then dispatched by post to Centre for Sustainable Aquatic Research (CSAR), Swansea, UK, to undertake post-preservation functional/bioremediation stability assessment.

### Statistical analysis

Statistical analysis of data was conducted using R software, using two- or three-factor analysis of variance allowing us to assess the differences between each treatment (i.e. medium; strain) and the data evolution in time. The analysis was conducted on the following variables: cell density, cell size and biovolume. When a significant difference was found, a post hoc Tukey test was used at a confidence level of 95%. The statistical interaction between factors was also assessed by the Spearman correlation test.

## Results

### Bioremediation using the algal consortium in a 2- and 600-L photobioreactor

**Fig. 1:**
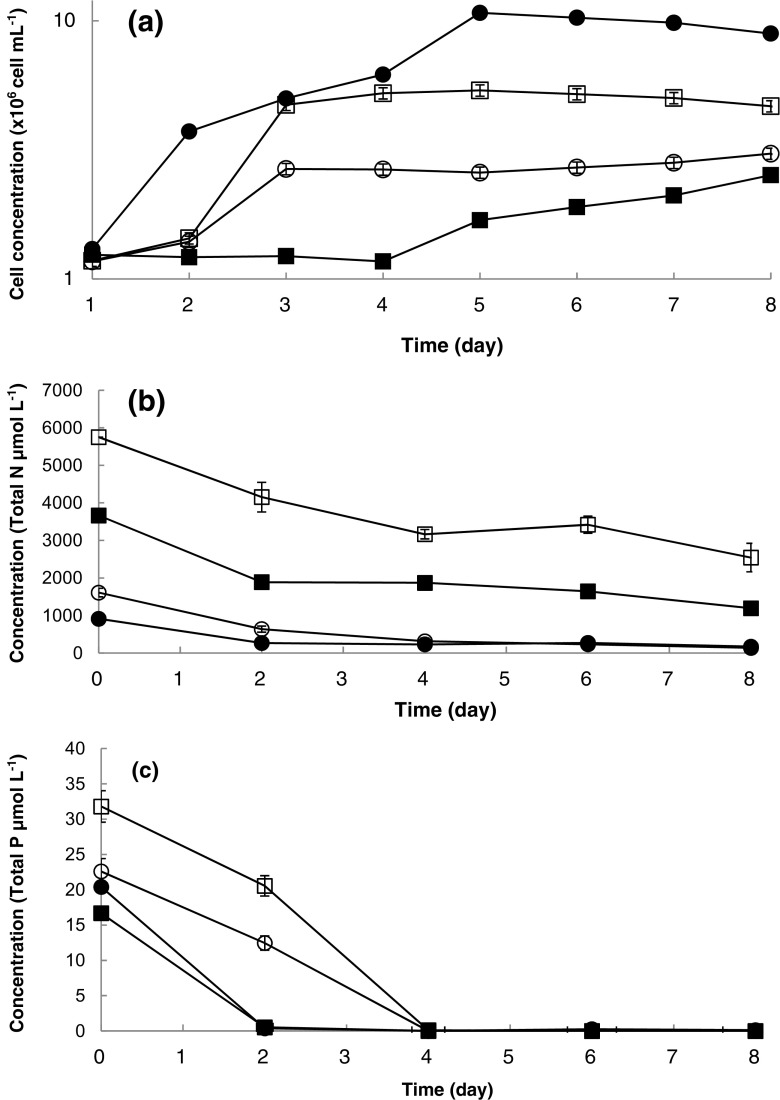
Growth and nutrient uptake of the ACCOMPLISH algal consortium in 250-mL flask under f/2 (*black circle*), 1% (*white circle*), 5% (*black square*) and 10% (*white square*) of effluent nutrients. **a** Cell density. **b** Bioremediation—removal of nitrogen. **c** Bioremediation—removal of phosphorous (error bars indicate the standard deviation, the number of independent replicates = 3)

The algal consortium was successfully grown on media supplemented with “waste nutrients” at 1, 5 and 10% (*v*/*v*) and control f/2 (Fig. 1a). Adaptation to the new nutrient regime was observed over the first 2 days under all waste dilutions tested and the best growth observed was in a medium supplemented with 10% waste effluent. The exponential growth rate in the 10% growth medium was 0.4 day^−1^, in comparison to 0.35 day^−1^ in freshwater f/2; however, the duration of the exponential phase was 3 days in the cultures grown on waste nutrients and 5 days in f/2. The growth observed in media supplemented with 1 and 5% of waste effluent was lower, and statistical analysis indicated that the medium formulation used had an impact of the growth estimated by cell density (*p* < 0.05) (Fig. 1a) (Table 2). The interaction between both factors was significant for both the cell density and the biovolume (*p* < 0.05), demonstrating that there was a significant difference in growth between both media; however, that difference was not the same over the time course of the experiment. Indeed, the cell density results indicated that there were no apparent differences between the two media at the beginning of the experiment, but there were differences after a few days of incubation. Furthermore, the post hoc Tukey test indicated that employing the control medium resulted in higher cell density and biovolume. Finally, the correlation test showed that the three variables (cell density, cell size and biovolume) were correlated, demonstrating that under all conditions tested that when a high cell density was observed, cells were larger and total biovolume higher (Table 2).

**Table 2:** Biovolume, cell concentration and cell size of algal consortium growing on different nutrient conditions and scale (± indicates the standard deviation, the number of independent replicates = 3)

Treatment	Biovolume (× 10^8^ μm^3^ mL^−1^)	Cell concentration (× 10^6^ cell mL^−1^)	Cell size (μm)
1% effluent (250 mL)	2.3 ± 0.3	2.7 ± 0.3	5.2
5% effluent (250 mL)	5.2 ± 0.5	3.6 ± 0.3	5.5
10% effluent (250 mL)	2.8 ± 0.1	7.7 ± 0.1	5.5
Control (f/2) (250 mL)	1.8 ± 0.2	5.5 ± 0.4	5.4
10% effluent (600 L)	4.8 ± 0.5	3.7 ± 0.1	7.7
Control (f/2) (600 L)	5.8 ± 0.5	6.5 ± 0.3	7.1

The nutrient uptake by the algal consortium is shown in Fig. 1b, c. Nitrogen was gradually removed from the media by the algal cultures under all the experimental conditions tested. Phosphate uptake was more rapid and by day 4, all the phosphorus in the media had been assimilated by the algal cultures under all nutrient conditions tested. The waste medium used had an impact on the nitrogen uptake rate (*p* < 0.05) and the results of post hoc tests (Fig. 1). The interaction between the concentration of both factors was significant for the cell density and the biovolume (*p* < 0.05) indicating that there was a significant difference in performance between media; however, that difference was not related to the time of sampling. The cell density results indicated that there were no apparent differences between culture nutrient uptake in the control and experimental culture media at the beginning of the experiment, but differences were observed after a few days of incubation, due to the adaptation to the media.

**Fig. 2:**
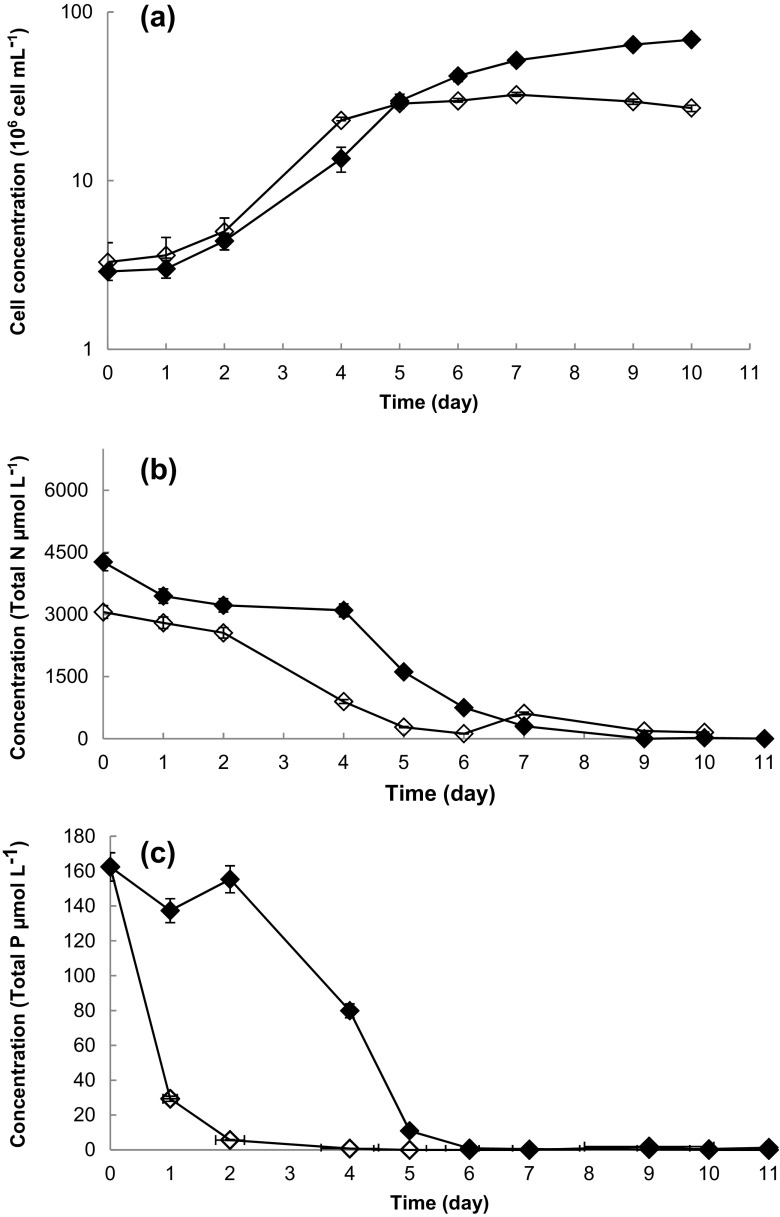
Growth and nutrient uptake of the ACCOMPLISH algal consortium in a 600-L PBR under (*black diamond*) f/2 and (white diamond) 10% of effluent nutrients. **a** Cell density. **b** Bioremediation—removal of nitrogen. **c** Bioremediation—removal of phosphorous (error bars indicate the standard deviation, the number of independent replicates = 3)

The algal consortium was successfully grown in PBR remediating a waste-based medium (Fig. 2a), (Table 2). The lag phase, adaptation to the reactor and nutrient conditions, was observed for the first 2 days for both control and waste nutrient conditions; the growth rate in this period was 0.1 day^−1^. The subsequent exponential phase lasted 4 days with a growth rate of 0.35 day^−1^ for the culture grown on AD waste nutrients. This was significantly different (*p* > 0.005) from the control (f/2) medium where the culture grew at a slower rate and entered stationary phase after 5–6 days of cultivation. The highest final cell density (3 × 10^7^ cells mL^−1^) was observed in the waste water-grown culture (Fig. 2a) (Table 2). Statistical analysis demonstrated that both the time of experiment and the medium used influence the three studied variables: cell density, cell size and biovolume (*p* < 0.05). Furthermore, the interaction between both factors was significant for the cell density and the biovolume (*p* < 0.05) indicating that there was a significant difference in culture growth between both media (Table 2); however, that difference was not dependent on the time of sampling. Indeed, the cell density data indicated that there were no apparent differences between adaptations in the two different media formulations at the beginning of the experiment, but there were differences after a few days of incubation. Furthermore, the post hoc Tukey test demonstrated that using the waste medium resulted in higher cell density and biovolume. Finally, the correlation test indicated that the three variables were correlated, demonstrating that at higher cell density, there were also bigger cells and thus greater total biovolume (Table 2).

The nitrogen uptake observed was not significantly different (*p* < 0.05) between waste and control (f/2) conditions (Fig. 2). By day 6, total nitrogen was remediated, i.e. removed from media by the algal consortium (significantly different (*p* < 0.05) than f/2 medium grown consortium). The phosphorus uptake for control and waste-based media growth conditions were significantly different (*p* < 0.05). The algal consortium grown on waste medium had slow phosphorus uptake during the first 3 days; after that, the phosphorus uptake accelerated. By day 6, all the phosphorus had been removed by the algal consortium showing rapid waste remediation, comparable to the control (freshwater f/2) treatment.

### Validation of bioremediation capacity of the cryopreserved algal consortium

On thawing of samples after 24 h and 3 weeks storage under liquid nitrogen, relatively rapid recovery and regrowth of the consortium were observed in all replicate cultures. Within 2–3 weeks, the cultures were comparable in density and appearance to the culture prior to cryopreservation. No overgrowth of the algal culture by the commensal bacteria was observed, and on microscopy, all replicates appeared to be effectively identical with respect to algal morphotypes present and were indistinguishable by light microscopy from the non-cryopreserved control samples that had been maintained in parallel under the standard cultivation regime.

**Fig. 3:**
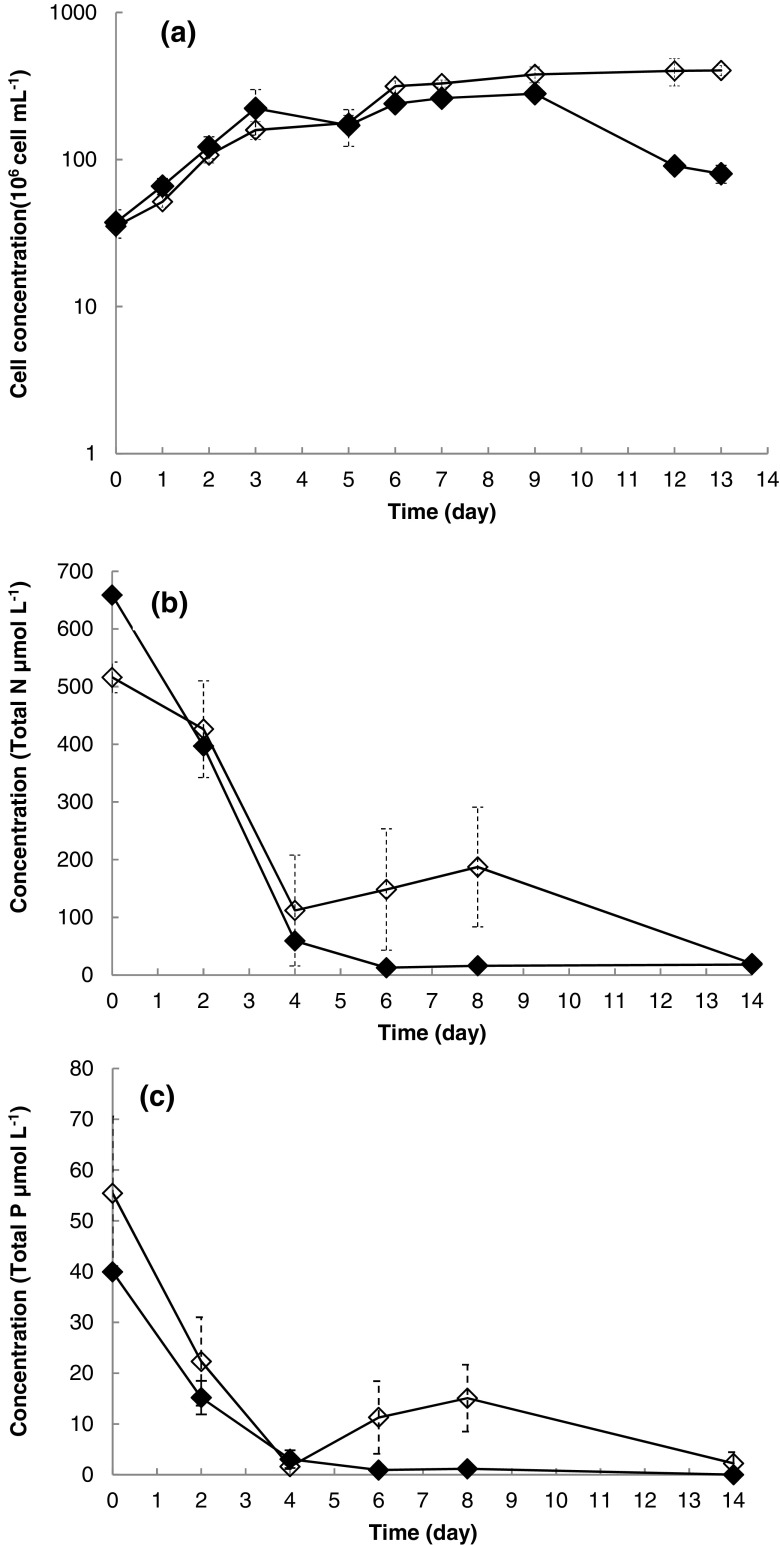
Growth and nutrient uptake for the ACCOMPLISH algal consortium before and after cryopreservation in flask (250 mL) system; *black diamond*—ACCOMPLISH mixed consortium before cryopreservation, *white diamond*—ACCOMPLISH mixed consortium after cryopreservation. **a** Cell density. **b** Bioremediation—removal of nitrogen (flask system). **c** Bioremediation—removal of phosphorous (*error bars* indicate the standard deviation, the number of independent replicates = 3)

On testing the growth of the two cultures, the non-cryopreserved algal consortium and the algal consortium after cryopreservation, over 13 days, good growth levels were observed (Fig. 3). No significant difference (*p* < 0.05) was observed between growth rates of the algal consortium before and after cryopreservation, which were 0.6 and 0.7 day^−1^, respectively (Fig. 3a). The nutrient remediation functionality (N and P uptake rate) was very similar in comparison with the previous experiment, and by day 4, 99% of the total nitrogen and total phosphorus were remediated by both of the cultures studied (Fig. 3b, c).

## Discussion

Conventional wastewater treatment technologies (e.g. anaerobic digestion) still have technical-economic limitations, mainly caused by their high energy requirement and relatively poor nutrient removal (de Godos et al. 2010). The development of algal technology for bioremediation applications has the potential to overcome these constraints as they can provide a system capable of high levels of nutrient removal, with the capacity to remediate a wide spectrum of waste nutrients by an environmentally friendly alternative to conventional methods (Boelee et al. 2011; Posadas et al. 2013; Polishchuk et al. 2015). The advantages of the use of mixed microalgal consortium have been the focus of recent research (Kerckhof et al. 2014; Mahapatra et al. 2014; Lahel et al. 2016). Furthermore, similar to the results of this study, high levels (above 90%) of remediation of inorganic nutrients have been reported in studies using both mixed natural or artificially formulated algal consortia (Fergola et al. 2007; Chinnasamy et al. 2010b; Renuka et al. 2013) . In this study, we have used a natural algal mixed consortium, where the dominant groups of algal species belong to either the Chlorophyceae or Trebouxiophyceae. Taxa belonging to these classes, particularly *Chlorella* and *Scenedesmus*, have been demonstrated to have high bioremediation potential (Chinnasamy et al. 2010a; 2012 Su et al.; Shene et al. 2016). However, *F. amphitricha* has not previously been reported in bioremediation studies (Tsarenko and John 2011).

The use of municipal waste nutrient source was one of the objectives of this study and the remediation of 4500 μmol L^−1^ (64 mg L^−1^) of N and 690 μmol L^−1^ (21 mg L^−1^) of P by a reduction of 99% was achieved using the algal consortium. Other research studies (Woertz et al. 2009; Chinnasamy et al. 2010b; Silva-Benavides and Torzillo 2012; Su et al. 2012) have reported comparable remediation capacities of 41–100% for N and 12–100% for phosphorus. However, this study has demonstrated that this is achievable at pilot-scale, rather than lab-scale (0.25–5 L) volume. The cultivation of the algal consortium with waste nutrient removal in a 600-L PBR in greenhouse reflected real environmental conditions (variable light, pH, nutrients availability, temperature) similar in any potential industrial site, rather than in laboratory. All these parameters influence the consortium behaviour and bioremediation capacity; however, although the remediation process in PBR was slightly longer than in the lab-scale experiment, as has previously been reported, the culture was more stable (Bordel et al. 2009). Overall, the scalability, nutrient removal capability and cell density of the algae produced demonstrated the potential of an integrated system capable of both bioremediation and the production of algal biomass.

In the biotechnological application of algae in phyco-remediation, as in any other industrial application, stability of the alga(e) and/or the mixed consortium is a pre-requisite for ensuring sustainable results/production. In case of successful waste remediation by algae, the main challenges are to maintain the long-term effectiveness and long-term homeostasis of the mixed consortium. (Gonçalves et al. 2016). The use of cryopreserved master stock cultures provides insurance for the sustainability of production. Furthermore, it provides practitioners with an option to ensure consistency in batch-based processes, where inoculum build-up may be initiated for each batch from the master stock culture. Cryopreservation is also an important tool for the Biological Resource Centre (BRC) community to hold patented strains, minimising risks associated with alternative procedures such as serial transfer and facilitating the exploitation of non-traditional biological resources (Stacey and Day 2014). Under the patent procedure, the strain deposited should retain the trait/capacity of commercial relevance. As part of the procedure, on the request of the depositor/owner of the strain, the cryopreserved samples may be revived, dispatched and re-tested for their biotechnological capabilities. There are numerous reports on the conservation of algae by cryopreservation for biotechnological or aquaculture use, highlighting the importance of minimising risks associated with losing, contaminating or the potential loss of traits by genetic drift (Cañavate and Lubińn 1995; Day et al. 2005; Rhodes et al. 2006). However, despite the importance of having evidence based on the retention of biotechnological potential, there are few commercially relevant reports in the literature. Hédoin et al. (2006) demonstrated the retention in capacity post-thaw of *Porphyridium cruentum* to produce zeaxanthin and beta-carotene and for the cyanobacterium *Planktothrix* to produce a cytotoxin. In addition, Nakanishi et al. (2012) have demonstrated that the chlorophyll content of *Nannochloropsis oculata* ST-4 and *Tetraselmis tetrathele* T-501 was not significantly changed after 15 years of cryostorage. More recently, Hipkin et al. (2014) reported the successful cryopreservation of the transgenic diatom *Thalassiosira pseudonana* CCAP 1085/23, which overexpressed a GFP-tagged nuclear localised protein, pre- and post-cryopreservation as a proxy for a biotechnological product. To date, there have been no previous reports on the cryopreservation of a mixed algal consortium. However, there are few reports on the retention of functionality of cryopreserved microbial consortia for environmental remediation including Augustynowicz et al. (2008) who demonstrated the capacity of a revived cryopreserved mixed bacterial community to degrade petroleum-derived environmental contaminants and Kerckhof et al. (2014) who successfully optimised the conservation of a methanotrophic co-culture (MOB), with potential for mitigation of greenhouse gas emissions, environmental pollutant removal and bioplastics production, as well as an oxygen-limited autotrophic nitrification/denitrification (OLAND) biofilm, with enhanced economic and ecological benefits for wastewater treatment. These consortia both retained good levels of functionality, although the preservation of the community structure (as determined by 16S rRNA gene sequencing) was incomplete. In our study community structure, beyond the presence of the key algal taxa, this was not determined, as functionality was the primary requirement.

In this study, we have confirmed the integrity of mixed consortium after the cryopreservation and demonstrated its capacity to bioremediate effluent effectively. This has significant implications to the algal biotechnology sector as a whole, where in reality, mixed/non-axenic cultivation will be a pre-requisite for future economic success. In addition, this paper provides a successful model of a procedure that has allowed the protection of intellectual property (IP) associated with a mixed consortium. This is in the vanguard of the BRC communities’ attempts to service the challenges associated with rapid developments in algal/protistan biotechnology.

In conclusion, microalgae, with their photoautotrophic capabilities, are able to uptake the waste nutrients using solar energy and carbon dioxide and can thus convert these nutrients to valuable biomass. This capability will, in the opinion of the authors, be a major component in a more integrated bioeconomy that will help to manage pollution worldwide, resulting from expansion of the global population and industrial activities. Underpinning these capabilities by guaranteeing the functional stability of conserved consortia is a key component in ensuring sustainability and long-term biotechnological exploitability.
